# A simulation study: Using dual ancillary variable to estimate population mean under stratified random sampling

**DOI:** 10.1371/journal.pone.0275875

**Published:** 2022-11-28

**Authors:** Sohaib Ahmad, Sardar Hussain, Uzma Yasmeen, Muhammad Aamir, Javid Shabbir, M. El-Morshedy, Afrah Al-Bossly, Zubair Ahmad

**Affiliations:** 1 Department of Statistics Abdul Wali Khan University Mardan Pakistan; 2 Department of Statistics, Quaid-i-azam University, Islamabad, Pakistan; 3 Institute of Molecular Biology and Biotechnology, The University of Lahore, Lahore, Pakistan; 4 Department of Mathematics, College of Science and Humanities in Al-Kharj, Prince Sattam Bin Abdulaziz University, Al-Kharj, Saudi Arabia; 5 Faculty of Science, Department of Mathematics, Mansoura University, Mansoura, Egypt; 6 Department of Statistics, Yazd University, Yazd, Iran; University of Sargodha, PAKISTAN

## Abstract

In this paper, we propose an improved ratio-in-regression type estimator for the finite population mean under stratified random sampling, by using the ancillary varaible as well as rank of the ancillary varaible. Expressions of the bias and mean square error of the estimators are derived up to the first order of approximation. The present work focused on proper use of the ancillary variable, and it was discussed how ancillary variable can improve the precision of the estimates. Two real data sets as well as simulation study are carried out to observe the performances of the estimators. We demonstrate theoretically and numerically that proposed estimator performs well as compared to all existing estimators.

## 1. Introduction

In sampling theory, appropriate use of the ancillary information may increase precision of the estimators. Numerous authors employed the ancillary information at the designing stage and at the estimation stage. One purpose of sample survey theory is to estimate unknown population parameters of the variable of intrest being studied, such as mean, median, proportion and variance etc. It is preferable to employ stratified random sampling scheme rather than simple random sampling when data is based on hetrogeneous population.

In stratified random sampling, we divide the diverse population into strata or groups which are non-overlapping, and sampling is carried out from each stratum separetely. Zaman and Kadilar [1] when the difference in variance across strata is significantly greater than the difference in variance within strata, stratification enhances efficiency of the estimates. Zaman and Kadilar [2] and Zaman [3] provided an efficient estimators of population mean using the auxiliary varaible in stratified random sampling. Zaman [4] proposed an efficient exponential type estimator for the population mean under stratified random sampling. Rather and Kadilar [5] introduced dual to ratio cum product type of exponential estimator under stratified random sampling. Mradula et al. [6] obtained an efficient estimation of population mean under stratified random smapling with linear cost function. Javed et al. [7] proposed a simulation based study for progressive estimation of population mean through traditional and non-traditional measures in stratified random sampling. Javed and Irfan [8] obtained a simulation based on new optimal estimators for population mean by using the dual auxiliary information in stratified random sampling. Yadav and Tailor [9] estimated a finite population mean using two auxiliary varaibles under stratified random sampling. Zaman and Bulut [10] proposed a modified regression estimator using robust regression methods and covaraince matrics in stratified sampling scheme. Kumar and Vishwakarma [11] proposed a generalized classes of regression-cum-ratio type estimators for population mean under stratified random sampling. Zahid et al. [12] provided a generalized class of estimators for sensitive varaible in the presence of measurment error and non-response under stratified random sampling. Some important references to the population mean under stratified random sampling using the auxiliary information include Aladag and Cingi [13], Grover and Kaur [14], Shabbir and Gupta [15], Khalid [16,17], Kadilar and Cingi [18,19], Koyuncu and Kadilar [20,21], Singh and Vishwakarma [22]. Recnetly Hussain et al. [23] proposed estimation of finite population distribution function with dual use of the anillary infromation under simple and stratified random sampling.

A unique idea for investigating more optimum estimators involving dual use of the ancillary information to deal with the stratified random sampling method has emerged recently. In this paper, we develop a new efficient estimator for finite population mean using dual ancillary variable under stratified random sampling.

Consider a finite population of distinct and identifiable units, Ѵ = $\left\{M_{1},M_{2},\ldots ,M_{Ŋ}\right\}$ of size Ŋ, are separated into L homogeneous strata of size Ŋ_*h*_(h = 1, 2,…, L), such that $\sum_{h=1}^{L}{Ŋ}_{h}=Ŋ$. A simple random sample of size *n*_*h*_ is drawn without replacement from the *h*^*th*^ stratum such that $\sum_{h=1}^{L}n_{h}=n$.

Let y, x and *r*_*x*_ be the study, auxiliary and rank of the auxiliary variables respectively, assuming values$y_{h_{i}},x_{h_{i}}$ and $r_{xh_{i}}$ for the *i*^*th*^ unit in the *h*^*th*^ stratum. Let the stratum means be ${\bar{Y}}_{h}=\sum_{i=1}^{{Ŋ}_{h}}\frac{y_{h_{i}}}{{Ŋ}_{h}},\;{\bar{X}}_{h}=\sum_{i=1}^{{Ŋ}_{h}}\frac{x_{h_{i}}}{{Ŋ}_{h}},\;{\bar{R}}_{xh}=\sum_{i=1}^{{Ŋ}_{h}}\frac{r_{xh_{i}}}{{Ŋ}_{h}}$ respectively. Let ${\bar{y}}_{st}=\sum_{h=1}^{L}{ɷ}_{h}{\bar{y}}_{h},\;{\bar{x}}_{st}=\sum_{h=1}^{L}{ɷ}_{h}{\bar{x}}_{h}$ and ${\bar{r}}_{xst}=\sum_{h=1}^{L}{ɷ}_{h}{\bar{r}}_{xh}$ be the sample means of y, x and *r*_*x*_ respectively across the strata, where ${\bar{y}}_{h}=\frac{1}{n_{h}}\sum_{i=1}^{n_{h}}y_{hi},{\bar{x}}_{h}=\frac{1}{n_{h}}\sum_{i=1}^{n_{h}}x_{hi}$ and ${\bar{r}}_{xh}=\frac{1}{n_{h}}\sum_{i=1}^{n_{h}}r_{xhi}$ are the sample stratum means, ${ɷ}_{h}=\frac{{Ŋ}_{h}}{Ŋ}$ are the known stratum weights. Let $\bar{Y}=\sum_{h=1}^{L}{ɷ}_{h}{\bar{Y}}_{h},\;\bar{X}=\sum_{h=1}^{L}{ɷ}_{h}{\bar{X}}_{h}$ and ${\bar{R}}_{x}=\sum_{h=1}^{L}{ɷ}_{h}{\bar{R}}_{x}$ are the population means of y, x and *r*_*x*_ respectively.

We also define the following error terms.

Let $\varepsilon_{0st}=\frac{{\bar{y}}_{st}-\bar{Y}}{\bar{Y}},\;\varepsilon_{1st}=\frac{{\bar{x}}_{st}-\bar{X}}{\bar{X}},\;\varepsilon_{2st}=\frac{{\bar{r}}_{xst}-{\bar{R}}_{x}}{{\bar{R}}_{x}}$ such that E(*ε*_*i*_) = 0, for (i = 0,1,2),

$$\mathrm{where},\;v_{rst}=\sum_{h=1}^{L}{ɷ}_{h}^{r+s+t}\frac{E[{\left({\bar{y}}_{h}-\bar{Y}\right)}^{r}{\left({\bar{x}}_{h}-\bar{X}\right)}^{s}{\left({\bar{r}}_{xh}-{\bar{R}}_{x}\right)}^{t}]}{{\bar{Y}}^{r}{\bar{X}}^{s}{{\bar{R}}_{x}}^{t}}$$

Using these notations, we have

$$E(\varepsilon_{0st}^{2})=\frac{\sum_{h=1}^{l}{ɷ}_{h}^{2}\lambda_{h}C_{yh}^{2}}{{\bar{Y}}^{2}}=v_{200},\;E(\varepsilon_{1st}^{2})=\frac{\sum_{h=1}^{l}{ɷ}_{h}^{2}\lambda_{h}C_{xh}^{2}}{{\bar{X}}^{2}}=v_{020},\;E(\varepsilon_{2st}^{2})=\frac{\sum_{h=1}^{l}{ɷ}_{h}^{2}\lambda_{h}C_{rxh}^{2}}{{\bar{R}}_{x}^{2}}=v_{002},$$

$${E(\varepsilon }_{0st}\varepsilon_{1st})=\frac{\sum_{h=1}^{l}{ɷ}_{h}^{2}\lambda_{h}S_{yxh}}{\bar{Y}\bar{X}}=v_{110},\;E(\varepsilon_{0st}\varepsilon_{2st})=\frac{\sum_{h=1}^{l}{ɷ}_{h}^{2}\lambda_{h}S_{yrxh}}{\bar{Y}{\bar{R}}_{x}}=v_{101},$$

$${E(\varepsilon }_{1st}\varepsilon_{2st})=\frac{\sum_{h=1}^{l}{ɷ}_{h}^{2}\lambda_{h}S_{xrxh}}{\bar{X}{\bar{R}}_{x}}=v_{011},\;\lambda_{h}=\left(\frac{1}{n_{h}}-\frac{1}{N_{h}}\right).$$

Let $C_{yh}=\frac{S_{yh}}{\bar{Y}},\;C_{xh}=\frac{S_{xh}}{\bar{X}}$, and $C_{rxh}=\frac{S_{rxh}}{{\bar{R}}_{x}}$, be the coefficients of variation of y, x and *r*_*x*_ and $\rho_{y_{h}x_{h}},\rho_{y_{h}rx_{h}}$, and $\rho_{x_{h}rx_{h}}$ be the population correlation coefficients between (*y*_*h*_, *x*_*h*_), (*y*_*h*_, *r*_*xh*_), and (*x*_*h*_, *r*_*xh*_) respectively in the *h*^*th*^ stratum. Let $s_{yh}^{2}=\frac{{\sum_{i=1}^{{Ŋ}_{h}}\left(y_{hi}-{\bar{y}}_{h}\right)}^{2}}{{Ŋ}_{h}-1},\;s_{xh}^{2}=\frac{{\sum_{i=1}^{{Ŋ}_{h}}\left(x_{hi}-{\bar{x}}_{h}\right)}^{2}}{{Ŋ}_{h}-1}$, and $s_{rxh}^{2}=\frac{{\sum_{i=1}^{{Ŋ}_{h}}\left({rx}_{hi}-{\bar{r}x}_{h}\right)}^{2}}{{Ŋ}_{h}-1}$, are the sample variances in the *h*^*th*^ stratum, where $s_{yh}=\sqrt{\frac{{\sum_{i=1}^{{Ŋ}_{h}}\left(y_{hi}-{\bar{y}}_{h}\right)}^{2}}{{Ŋ}_{h}-1}},\;s_{xh}=\sqrt{\frac{{\sum_{i=1}^{{Ŋ}_{h}}\left(x_{hi}-{\bar{x}}_{h}\right)}^{2}}{{Ŋ}_{h}-1}}$, and $s_{rxh}=\sqrt{\frac{{\sum_{i=1}^{{Ŋ}_{h}}\left({rx}_{hi}-{\bar{r}x}_{h}\right)}^{2}}{{Ŋ}_{h}-1}}$ are the sample standard deviation in the *h*^*th*^ stratum. Let $s_{yxh}=\frac{\sum_{i=1}^{{Ŋ}_{h}}\left(y_{hi}-{\bar{y}}_{h})(x_{hi}-{\bar{x}}_{h}\right)}{{Ŋ}_{h}-1},\;s_{yrxh}=\frac{\sum_{i=1}^{{Ŋ}_{h}}\left(y_{hi}-{\bar{y}}_{h})(r_{xhi}-{\bar{r}}_{xh}\right)}{{Ŋ}_{h}-1}$, and $s_{xrxh}=\frac{\sum_{i=1}^{{Ŋ}_{h}}\left(x_{hi}-{\bar{x}}_{h})(r_{xhi}-{\bar{r}}_{xh}\right)}{{Ŋ}_{h}-1}$, are the covariance between their respective subscripts.

The rest of the paper is arranged as follows: In Section 2, literature review of various estimators under stratified random sampling is introduced. In Section 3, we proposed estimator for estimating finite population mean under stratified random sampling using dual ancillary variables are defined. In Section 4, theoretical comparison is conducted to assess the performances of the estimators. In Section 5 and 6, numerical investigation and data description are given. Monte Carlo simulation is concluded in Section 7. Discussions of the numerical results are given in Section 8. Finally, concluding remarks are discussed in Section 9.

## 2. Literature review

This section studies various stratified estimators that are available in the literature:

- The traditional mean estimator in stratified random sampling is:
$${\hat{\bar{Y}}}_{\left(st\right)}=\sum_{h=1}^{L}{ɷ}_{h}{\bar{y}}_{h}.$$
  The variance of ${\hat{\bar{Y}}}_{st}$, is given by
$$\mathrm{Var}({\hat{\bar{Y}}}_{\left(st\right)})={\bar{Y}}^{2}v_{200}$$ (1)
- Cochran [24], suggested the traditional ratio ${\hat{\bar{Y}}}_{R(st)}$ estimator:
$${\hat{\bar{Y}}}_{R_{\left(st\right)}}={\bar{y}}_{\left(st\right)}\left(\frac{\bar{X}}{{\bar{x}}_{\left(st\right)}}\right)$$ (2)
  The bias and MSE of ${\hat{\bar{Y}}}_{R}$, are given by:
$$Bias({\hat{\bar{Y}}}_{R(st)})=\bar{Y}(v_{020}-v_{110}),$$
  and
$$MSE({\hat{\bar{Y}}}_{R(st)})≅{\bar{Y}}^{2}(v_{200}+v_{020}-2v_{110})$$ (3)
- Murthy [25] suggested the usual product estimator ${\hat{\bar{Y}}}_{P(st)}$, which is given by:
$${\hat{\bar{Y}}}_{P(st)}={\bar{y}}_{\left(st\right)}\left(\frac{{\bar{x}}_{\left(st\right)}}{\bar{X}}\right)$$ (4)
  The bias and MSE of ${\hat{\bar{Y}}}_{P(st)}$, are given by:
$$Bias({\hat{\bar{Y}}}_{P(st)})=\bar{Y}v_{110},$$
  and
$$MSE\left({\hat{\bar{Y}}}_{P(st)}\right)≅{\bar{Y}}^{2}(v_{200}+v_{020}+2v_{110})$$ (5)
- Bahl and Tuteja [26] suggested the following estimators:
$${\hat{\bar{Y}}}_{BT,R(st)}={\bar{y}}_{\left(st\right)}exp(\frac{\bar{X}-{\bar{x}}_{\left(st\right)}}{\bar{X}+{\bar{x}}_{\left(st\right)}}),$$ (6)
  and
$${\hat{\bar{Y}}}_{BT,P(st)}={\bar{y}}_{\left(st\right)}exp\left(\frac{{\bar{x}}_{\left(st\right)}-\bar{X}}{{\bar{x}}_{\left(st\right)}+\bar{X}}\right)$$ (7)
  The biases and MSEs of ${\hat{\bar{Y}}}_{BT,R(st)}$, and ${\hat{\bar{Y}}}_{BT,P(st)}$, are given by:
$$Bias\left({\hat{\bar{Y}}}_{BT,R(st)}\right)≅\bar{Y}(\frac{3}{8}v_{020}-\frac{1}{2}v_{110}),$$
$$MSE\left({\hat{\bar{Y}}}_{BT,R(st)}\right)=\frac{{\bar{Y}}^{2}}{4}(4v_{200}+v_{020}-4v_{110}).$$ (8)
  and
$$Bias\left({\hat{\bar{Y}}}_{BT,P(st)}\right)=\bar{Y}(\frac{1}{2}v_{110}-\frac{1}{8}v_{020}),$$
$$MSE\left({\hat{\bar{Y}}}_{BT,P(st)}\right)=\frac{{\bar{Y}}^{2}}{4}(4v_{200}+v_{020}+4v_{110}).$$ (9)
- The difference estimator ${\hat{\bar{Y}}}_{dif}$, is given by:
$${\hat{\bar{Y}}}_{dif(st)}={\bar{y}}_{\left(st\right)}+d(\bar{X}-{\bar{x}}_{\left(st\right)}),$$ (10)
  where d is an appropriate constant. The minimum variance of ${\hat{\bar{Y}}}_{dif(st)}$ at the optimal worth $d_{opt}=\frac{\bar{Y}v_{110}}{\bar{X}v_{020}}$, is given as:
$${Var({\hat{\bar{Y}}}_{dif(st)})}_{min}=\frac{{\bar{Y}}^{2}(v_{200}v_{020}-v_{110})}{v_{020}},$$ (11)
- Rao [27], proposed the following estimator:
$${\hat{\bar{Y}}}_{R,D(st)}=Q_{1}{\bar{y}}_{\left(st\right)}+Q_{2}(\bar{X}-{\bar{x}}_{\left(st\right)}),$$ (12)
  where *Q*_1_ and *Q*_2_ are constants.
  The bias and MSE of ${\hat{\bar{Y}}}_{R,D(st)}$, given by:
$$Bias({\hat{\bar{Y}}}_{R,D(st)})=\bar{Y}(Q_{1}-1),$$
  and
$$MSE({\hat{\bar{Y}}}_{R,D(st)})={\bar{Y}}^{2}–2Q_{1}{\bar{Y}}^{2}+Q_{1}^{2}{\bar{Y}}^{2}+Q_{1}^{2}{\bar{Y}}^{2}v_{200}‐2Q_{1}Q_{2}\bar{Y}\bar{X}v_{110}+Q_{2}^{2}{\bar{X}}^{2}v_{020}.$$
  The optimum values of *Q*_1_ and *Q*_2_ are given by:
$${Q_{1}}_{opt}=\frac{v_{020}}{\left(v_{020}v_{200}-{v_{110}}^{2}+v_{020}\right)},$$
  and
$${Q_{2}}_{opt}=\frac{\bar{Y}v_{110}}{\bar{X}(v_{200}v_{020}-{v_{110}}^{2}+v_{020})}.$$
  The minimum MSE of ${\hat{\bar{Y}}}_{R,D(st)}$ is given by:
$${MSE({\hat{\bar{Y}}}_{R,D(st)})}_{min}=\frac{{\bar{Y}}^{2}(v_{200}v_{020}-v_{101}^{2})}{\left(v_{200}v_{020}-{v_{110}}^{2}+v_{020}\right)}.$$ (13)
- The suggested estimator by Singh et al. [28]:
$${\hat{\bar{Y}}}_{Singh(st)}={\bar{y}}_{\left(st\right)}exp(\frac{a(\bar{X}-{\bar{x}}_{\left(st\right)})}{a(\bar{X}+{\bar{x}}_{\left(st\right)})+2b}),$$ (14)
  For a = 1, and b = 0,
  The bias and MSE of ${\hat{\bar{Y}}}_{Singh}$, is given by:
$$Bias\left({\hat{\bar{Y}}}_{Singh(st)}\right)=\bar{Y}\left(\frac{3}{8}v_{020}-\frac{1}{2}v_{110}\right),$$
  and
$$MSE({\hat{\bar{Y}}}_{S(st)})≅\frac{{\bar{Y}}^{2}}{4}(4v_{200}+v_{020}-4v_{110}).$$ (15)
- The suggested estimator of Grover and Kaur [29], is given as:
$${\hat{\bar{Y}}}_{Gk(st)}=\left\{Z_{1}{\bar{y}}_{\left(st\right)}+Z_{2}(\bar{X}-{\bar{x}}_{\left(st\right)})\right\}exp(\frac{a(\bar{X}-\bar{x})}{a(\bar{X}+\bar{x})+2b}).$$ (16)
  Where *Z*_1_ and *Z*_2_ are unknown constants. For a = 1 and b = 0
  The bias and MSE of ${\hat{\bar{Y}}}_{Gk(st)}$ are given by:
$$Bias({\hat{\bar{Y}}}_{Gk(st)})=\bar{Y}(Z_{1}-1)+\frac{3}{8}Z_{1}\bar{Y}+\frac{1}{2}Z_{2}\bar{X}v_{020}-\frac{1}{2}\bar{Y}v_{110}Z_{1},$$
  And
$${MSE({\hat{\bar{Y}}}_{Gk(st)})}_{min}=(Z_{1}-1){\bar{Y}}^{2}+Z_{1}^{2}{\bar{Y}}^{2}(v_{200}+v_{020}-2v_{110})+Z_{2}^{2}{\bar{X}}^{2}v_{020}‐2Z_{1}{\bar{Y}}^{2}(\frac{3}{8}v_{020}-\frac{1}{2}v_{110})–2Z_{2}\bar{Y}\bar{X}(\frac{1}{2}v_{020})‐2Z_{1}Z_{2}\bar{Y}\bar{X}(v_{110}-v_{020})$$
$${MSE({\hat{\bar{Y}}}_{Gk(st)})}_{min}≅Z_{2}^{2}{\bar{X}}^{2}v_{020}+Z_{1}^{2}{\bar{Y}}^{2}v_{200}+2Z_{1}Z_{2}\bar{Y}\bar{X}v_{020}-2Z_{1}Z_{2}\bar{Y}\bar{X}v_{110}+{\bar{Y}}^{2}–2Z_{1}{\bar{Y}}^{2}+Z_{1}^{2}{\bar{Y}}^{2}-2Z_{1}{\bar{Y}}^{2}v_{110}-Z_{2}\bar{Y}\bar{X}v_{020}-2Z_{1}^{2}{\bar{Y}}^{2}v_{110}-\frac{3}{4}Z_{1}{\bar{Y}}^{2}v_{020}+Z_{1}^{2}{\bar{Y}}^{2}v_{020}.$$ (17)
  The optimum values of *Z*_1_ and *Z*_2_ are given as:
$$Z_{1(opt)}=\frac{v_{020}(v_{020}-8)}{8(-v_{200}v_{020}-{v_{110}}^{2}-v_{020})},$$
$$Z_{2(opt)}=\frac{\bar{Y}(v_{020}^{2}-\vartheta_{020}v_{020}+4v_{200}v_{020}-4{v_{110}}^{2}-4v_{020}+8v_{110})}{8\bar{X}(v_{200}v_{020}-{v_{110}}^{2}+v_{020})},$$
  The minimal MSE of ${\hat{\bar{Y}}}_{Gk(st)}$, are:
$${{\hat{\bar{Y}}}_{GK(st)}}_{min}=\frac{{\bar{Y}}^{2}}{64}\left(64-16v_{020}-\frac{v_{020}{\left(-8+v_{020}\right)}^{2}}{{v_{020}}_{(1+v_{200})-v_{110}^{2}}}\right)$$ (18)
- Ahmad et al. [30] proposed an improved estimator ${\hat{\tilde{Y}}}_{Pr_{st}}$, is given by:
$${\hat{\tilde{Y}}}_{Pr_{st}}=\left\{Q_{5}{\hat{\tilde{Y}}}_{st}+Q_{6}(\frac{\tilde{X}-{\hat{\tilde{X}}}_{st}}{\tilde{X}})+Q_{7}(\frac{\tilde{Z}-{\hat{\tilde{Z}}}_{st}}{\tilde{Z}})\right\}exp(\frac{a(\tilde{X}-{\hat{\tilde{X}}}_{st})}{a(\tilde{X}+{\hat{\tilde{X}}}_{st})+2b}).$$ (19)
  Where *Q*_*i*_(i = 5,6,7) are constants.
  The bias and MSE of ${\hat{\tilde{Y}}}_{Pr_{st}}$, are given by:
$$B({\hat{\tilde{Y}}}_{Pr_{st}})≅\tilde{Y}(Q_{5}-1)+\frac{3}{8}\theta^{2}Q_{5}\tilde{Y}V_{020}+\frac{1}{2}\theta Q_{6}V_{020}-\frac{1}{2}\theta Q_{5}\tilde{Y}V_{110}+\frac{1}{2}\theta V_{011},$$
  and
$$MSE({\hat{\tilde{Y}}}_{Pr_{st}})≅{\tilde{Y}}^{2}(Q_{5}-1{)}^{2}+Q_{5}^{2}{\tilde{Y}}^{2}V_{200}+Q_{6}V_{020}+Q_{7}V_{002}+\theta^{2}Q_{5}^{2}{\tilde{Y}}^{2}V_{020}-\theta Q_{6}\tilde{Y}V_{020}+2\theta \tilde{Y}V_{020}-\frac{3}{4}\theta^{2}Q_{5}{\tilde{Y}}^{2}V_{020}+\theta Q_{5}{\tilde{Y}}^{2}V_{110}-2\theta Q_{5}^{2}{\tilde{Y}}^{2}V_{110}-2Q_{5}Q_{6}\tilde{Y}V_{110}-2Q_{5}Q_{7}\tilde{Y}V_{101}-\theta Q_{7}\tilde{Y}V_{011}+2\theta Q_{5}Q_{7}\tilde{Y}V_{011}-2Q_{6}Q_{7}V_{011}.$$ (20)
  The optimum values of *Q*_5_, *Q*_6_ and *Q*_7_ are given by:
$$Q_{5(opt)}=\frac{8-\theta^{2}V_{020}}{8\{1+V_{200}(1-R_{y.xz}^{2})\}},$$
$$Q_{6(opt)}=\frac{\tilde{Y}[\begin{array}{ll} & \theta^{3}V_{020}^{3/2}(R_{xz}^{2}-1)+V_{200}^{1/2}(-8+\theta^{2}V_{020})(R_{yx}-R_{xz}R_{yz})\\ & +4\theta V_{020}^{1/2}(R_{xz}^{2}-1)\{-1+V_{200}(1-R_{y.xz}^{2})\} \end{array}]}{8V_{020}^{1/2}(R_{xz}^{2}-1)\{-1+V_{200}(1-R_{y.xz}^{2})\}},$$
$$Q_{7(opt)}=\frac{\tilde{Y}V_{200}^{1/2}(8-\theta^{2}V_{020})(R_{yx}-R_{xz}R_{yz})}{8V_{020}^{1/2}(R_{xz}^{2}-1)\{-1+V_{200}(1-R_{y.xz}^{2})\}}.$$
  The minimum MSE of ${\hat{\tilde{Y}}}_{Pr_{st}}$ at optimum values of *Q*_5_, *Q*_6_, and *Q*_7_ are given by:
$$MSE({\hat{\tilde{Y}}}_{Pr_{st}})≅\frac{{\tilde{Y}}^{2}\{64V_{200}(1-R_{y.xz}^{2})-\theta^{4}V_{020}^{2}-16\theta^{2}V_{020}V_{200}(1-R_{y.xz}^{2})\}}{64\{1+V_{200}(1-R_{y.xz}^{2})\}},$$ (21)
  where $R_{y.xz}^{2}=\left(\frac{V_{110}^{2}V_{002}+V_{101}^{2}V_{020}-2V_{101}V_{110}V_{011}}{V_{200}(V_{020}V_{002}-V_{011}^{2})}\right)$.

## 3. Proposed estimator

Suitable use of the ancillary information may improve the precision of estimators both at the design and estimation stage. The rank of the ancillary variable is correlated with the study variable when the correlation among the study and ancillary variable is strong. In literature, dual use of ancillary variable has been rarely attempted, therefore we motivated towards it. The principal advantage of our proposed ratio-in-regression type estimator under stratified random sampling is that it is more flexible, efficient than the existing estimators. Taking motivation from Ahmad et al. [30], we propose ratio-in-regression type exponential estimator for estimating the population mean under stratified random sampling.


$${\hat{\bar{Y}}}_{ss(st)}=Q_{11}{\bar{y}}_{\left(st\right)}+Q_{12}(\bar{X}-{\bar{x}}_{\left(st\right)})exp\left(\frac{\bar{X}-{\bar{x}}_{\left(st\right)}}{\bar{X}+{\bar{x}}_{\left(st\right)}}\right)+Q_{13}({\bar{R}}_{x}-{\bar{r}}_{x(st)})exp\left(\frac{{\bar{R}}_{x}-{\bar{r}}_{x(st)}}{{\bar{R}}_{x}+{\bar{r}}_{x(st)}}\right)$$ (22)

Where *Q*_11_, *Q*_12_ and *Q*_13_ are unknown constants.

Solving ${\hat{\bar{Y}}}_{ss(st)}$ given in Eq (22),

$${\hat{\bar{Y}}}_{ss(st)}=Q_{11}\bar{Y}(1+\varepsilon_{0st})-Q_{12}\bar{X}\varepsilon_{1st}(1-\frac{1}{2}\varepsilon_{1st}+\frac{3}{8}\epsilon_{1st}^{2})‐Q_{13}{\bar{R}}_{x}\varepsilon_{2st}(1-\frac{1}{2}\varepsilon_{2st}+\frac{3}{8}\varepsilon_{2st}^{2})$$

$${\hat{\bar{Y}}}_{ss(st)}-\bar{Y}=(Q_{11}-1)\bar{Y}+Q_{11}\bar{Y}\varepsilon_{0st}-Q_{12}\bar{X}(\varepsilon_{1st}-\frac{1}{2}\varepsilon_{1st}^{2})-Q_{13}{\bar{R}}_{x}(\varepsilon_{2st}-\frac{1}{2}\varepsilon_{2st}^{2})$$ (i)

$$Bias\left({\hat{\bar{Y}}}_{ss(st)}\right)=(Q_{11}-1)\bar{Y}+\frac{1}{2}\bar{X}Q_{12}v_{020}+\frac{1}{2}Q_{13}{{\bar{R}}_{x}v}_{002}$$

Simplify Eq (i), we have

$$MSE({\hat{\bar{Y}}}_{ss(st)})={\left(Q_{11}-1\right)}^{2}{\bar{Y}}^{2}+Q_{11}^{2}{\bar{Y}}^{2}\epsilon_{0st}^{2}+Q_{12}^{2}{\bar{X}}^{2}\epsilon_{1st}^{2}+Q_{13}^{2}\bar{R}x^{2}\epsilon_{2st}^{2}+2\;Q_{11}(Q_{11}-1){\bar{Y}}^{2}\varepsilon_{0st}‐\;2\;(Q_{11}-1)Q_{12}\bar{Y}\bar{X}(\varepsilon_{1st}-\frac{1}{2}\varepsilon_{1st}^{2})-2\;(Q_{11}-1)Q_{13}{\bar{Y}\bar{R}}_{x}(\varepsilon_{2st}-\frac{1}{2}\varepsilon_{2st}^{2})-2\;Q_{11}Q_{12}\bar{Y}\bar{X}(\varepsilon_{0st}\varepsilon_{1st})-2\;Q_{11}Q_{13}{\bar{Y}\bar{R}}_{x}(\varepsilon_{0st}\varepsilon_{2st})-2\;Q_{12}Q_{13}{\bar{Y}\bar{R}}_{x}\varepsilon_{1st}\varepsilon_{2st}$$

$$={\left(Q_{11}-1\right)}^{2}{\bar{Y}}^{2}+Q_{11}^{2}{\bar{Y}}^{2}v_{200}+Q_{12}^{2}{\bar{X}}^{2}v_{020}+Q_{13}^{2}\bar{R}x^{2}v_{002}+2(Q_{11}Q_{12}-Q_{13})\bar{Y}\bar{X}\frac{v_{020}}{2}+22(Q_{11}Q_{13}-Q_{13}){\bar{Y}\bar{R}}_{x}\frac{v_{002}}{2}-2Q_{11}Q_{12}\bar{Y}\bar{X}v_{110}-2\;Q_{11}Q_{13}{\bar{Y}\bar{R}}_{x}v_{101}+2\;Q_{12}\;Q_{13}{\bar{X}\bar{R}}_{x}v_{011}$$

$$MSE({\hat{\bar{Y}}}_{ss(st)})={\left(Q_{11}-1\right)}^{2}{\bar{Y}}^{2}+Q_{11}^{2}{\bar{Y}}^{2}v_{200}+Q_{12}^{2}{\bar{X}}^{2}v_{020}+Q_{13}^{2}\bar{R}x^{2}v_{002}‐2Q_{12}\bar{Y}\bar{X}\frac{v_{020}}{2}-2Q_{13}{\bar{Y}\bar{R}}_{x}\frac{v_{002}}{2}+2Q_{11}Q_{12}\bar{Y}\bar{X}(\frac{v_{020}}{2}-v_{110})+2Q_{11}Q_{13}{\bar{Y}\bar{R}}_{x}(\frac{v_{002}}{2}-v_{101})+2Q_{12}Q_{13}\bar{X}\bar{R}x^{2}v_{011}$$ (23)

The optimum values of *Q*_11_, *Q*_12_ and *Q*_13_ are given by:

$$Q_{11(opt)}=\frac{(2\lambda \frac{{v_{200}}^{1/2}}{\lambda^{1/2}})(S_{11}+B_{11})+\lambda (T+\frac{v_{020}}{\lambda })-4}{4v_{200}\bar{Y}(R_{yx}^{2}-1)+4{v_{200}}^{1/2}(S_{11}+P_{11})+\lambda (T+\frac{v_{020}}{\lambda })-4},$$

$$Q_{12(opt)}=\frac{\frac{{v_{200}}^{1/2}}{\lambda^{1/2}}\bar{Y}[2v_{200}^{1/2}\lambda^{1/2}\{\frac{v_{002}^{1/2}}{\lambda^{1/2}}S_{11+}\frac{v_{020}^{1/2}}{\lambda^{1/2}}(\frac{v_{101}^{2}}{v_{200}v_{002}})\}{v_{002}}^{1/2}{v_{020}}^{1/2}D_{11}+4S_{11}]}{\left[\frac{{v_{020}}^{1/2}}{\lambda^{1/2}}\bar{X}(1-\frac{v_{101}^{2}}{v_{200}v_{002}})\{4v_{200}(R_{yx}^{2}-1)+\lambda {v_{200}}^{1/2}(S_{11}+B_{11})+\lambda (T+\frac{v_{020}}{\lambda })-4\}\right]},$$

$$Q_{13(opt)}=\frac{\frac{{v_{200}}^{1/2}}{\lambda^{1/2}}\bar{Y}[2{v_{200}}^{1/2}\lambda^{1/2}\{\frac{{v_{002}}^{1/2}}{\lambda^{1/2}}S_{11+}\frac{{v_{020}}^{1/2}}{\lambda^{1/2}}(\frac{\vartheta_{101}^{2}}{v_{200}v_{002}})\}+4S_{11}]}{\left[\frac{v_{020}^{1/2}}{\lambda^{1/2}}{\bar{R}}_{x}(1-\frac{v_{101}^{2}}{v_{200}v_{002}})\{4v_{200}(R_{yx}^{2}-1)+\lambda {v_{200}}^{1/2}(S_{11}+B_{11})+\lambda (T+\frac{v_{020}}{\lambda })-4\}\right]},$$

where

$$D_{11}=\frac{v_{110}}{\sqrt{v_{200}}\sqrt{v_{020}}}-\frac{v_{101}}{\sqrt{v_{200}}\sqrt{v_{002}}},\;R_{y.xz}^{2}=(\frac{v_{110}^{2}v_{002+v_{101}^{2}v_{020}-2v_{101}v_{110}v_{011}}}{v_{200}{}_{\left(v_{020}v_{002}-v_{110}^{2}\right)}}),$$

$$T=\frac{{\left\{\frac{v_{002}^{1/2}}{\lambda^{1/2}}-\frac{v_{020}^{1/2}}{\lambda^{1/2}}(\frac{v_{011}}{\sqrt{v_{020}}\sqrt{v_{002}}})\right\}}^{2}}{1-\frac{v_{011}^{2}}{v_{020}v_{002}}},\;S_{11}=\frac{\frac{v_{020}^{1/2}}{\lambda^{1/2}}(\frac{v_{101}}{\sqrt{v_{200}}\sqrt{v_{002}}}-\frac{v_{011}}{\sqrt{v_{020}}\sqrt{v_{002}}}-\frac{v_{110}}{\sqrt{v_{200}}\sqrt{v_{020}}})}{1-\frac{v_{011}^{2}}{v_{020}v_{002}}},$$

$$P_{11}=\frac{{\left\{\frac{v_{101}}{\sqrt{v_{200}}\sqrt{v_{002}}}(\frac{{v_{200}}^{1/2}}{\lambda^{1/2}})-\frac{v_{110}}{\sqrt{v_{020}}\sqrt{v_{002}}}(\frac{v_{002}^{1/2}}{\lambda^{1/2}})\right\}}^{2}}{1-\frac{v_{011}^{2}}{v_{020}v_{002}}},\;B_{11}=\frac{\frac{v_{002}^{1/2}}{\lambda^{1/2}}(\frac{v_{110}}{\sqrt{v_{200}}\sqrt{v_{002}}}-\frac{v_{010}}{\sqrt{v_{020}}\sqrt{v_{002}}}-\frac{v_{101}}{\sqrt{v_{200}}\sqrt{v_{002}}})}{1-\frac{v_{011}^{2}}{v_{020}v_{002}}}.$$

Putting the optimum values of *Q*_11_, *Q*_12_ and *Q*_13_ in (23), we get the minimal mean square error of ${\hat{\bar{Y}}}_{ss(st)}$, given by:

$${MSE({\hat{\bar{Y}}}_{ss(st)})}_{min}=\frac{{\bar{Y}}^{2}v_{200}\{T(\lambda )-B_{11}(\lambda )+v_{020}+4(R_{yx}^{2}-1)\}}{\left\{\lambda (4\frac{v_{200}}{\lambda }(R_{yx}^{2}-1)+4\frac{v_{020}^{1/2}}{\lambda^{1/2}}(S_{11}+B_{11})+T+\frac{v_{020}}{\lambda }-4)\right\}}.$$ (24)

## 4. Theoretical comparision

In this section, the theoretical comparison of the suggested estimator with existing estimators is considered:

- By taking (1) and (24),
$${MSE({\hat{\bar{Y}}}_{ss(st)})}_{min}<Var({\hat{\bar{Y}}}_{\left(st\right)})\;if$$
$$Var({\hat{\bar{Y}}}_{\left(st\right)})-{MSE({\hat{\bar{Y}}}_{ss(st)})}_{min}>0$$
$$\frac{{\bar{Y}}^{2}v_{200}[\frac{{Ɲ}_{11}}{\sqrt{\lambda (\frac{1}{2})}}\left[{Ɲ}_{13}\right]]}{\lambda [\frac{{Ɲ}_{12}}{\sqrt{\lambda (\frac{1}{2})}}]}>0$$
  where
$${Ɲ}_{11}=(T-4)\lambda +R_{yx}^{2}v_{200}+v_{020}-4v_{200}\sqrt{\lambda (\frac{1}{2})}+4\sqrt{v_{020}}\lambda (S_{11}+B_{11}),$$
$${Ɲ}_{12}=((T-4)\lambda +4R_{yx}^{2}v_{200}+v_{020}-4v_{200})\sqrt{\lambda (\frac{1}{2})}+4\sqrt{v_{020}}\lambda (S_{11}+B_{11}),$$
$${Ɲ}_{13}=T(\lambda )-B_{11}\lambda +v_{020}+4R_{yx}^{2}-4$$
  By taking (3) and (24),
$${MSE({\hat{\bar{Y}}}_{ss(st)})}_{min}<MSE({\hat{\bar{Y}}}_{R(st)})\;if$$
$$MSE({\hat{\bar{Y}}}_{R(st)})-{MSE({\hat{\bar{Y}}}_{ss(st)})}_{min}>0$$
$$\frac{{\bar{Y}}^{2}[\frac{\left(-v_{020}+2v_{110}-v_{200})\lambda ({Ɲ}_{11}\right)}{\sqrt{\lambda (\frac{1}{2})}}+v_{200}\left[{Ɲ}_{13}\right]]}{\lambda [\frac{{Ɲ}_{12}}{\sqrt{\lambda (\frac{1}{2})}}]}>0$$
- By taking (5) and (24),
$${MSE({\hat{\bar{Y}}}_{ss(st)})}_{min}<MSE({\hat{\bar{Y}}}_{P(st)})\;if$$
$$MSE({\hat{\bar{Y}}}_{P(st)})‐{MSE({\hat{\bar{Y}}}_{ss(st)})}_{min}>0.$$
$$\frac{{\bar{Y}}^{2}[\frac{\left(-v_{020}+2v_{110}-v_{200})\lambda ({Ɲ}_{14}\right)}{\sqrt{\lambda (\frac{1}{2})}}+v_{200}\left[{Ɲ}_{13}\right]]}{\lambda [\frac{{Ɲ}_{14}}{\sqrt{\lambda (\frac{1}{2})}}]}>0$$
  Where
$${Ɲ}_{14}=(T-4)+v_{020}-4v_{200}\sqrt{\lambda (\frac{1}{2})}+4\sqrt{v_{020}}\lambda (S_{11}+B_{11}).$$
- By taking (8) and (24),
$${MSE({\hat{\bar{Y}}}_{ss(st)})}_{min}<MSE({\hat{\bar{Y}}}_{BT,R(st)})\mathrm{if}$$
$$MSE({\hat{\bar{Y}}}_{BT,R(st)})‐{MSE({\hat{\bar{Y}}}_{ss(st)})}_{min}>0$$
$$\frac{{\bar{Y}}^{2}[\frac{\left(\frac{1}{4}v_{020}+v_{110}-v_{200})\lambda ({Ɲ}_{12}\right)}{\sqrt{\lambda (\frac{1}{2})}}-v_{200}\left[{Ɲ}_{13}\right]]}{\lambda [\frac{{Ɲ}_{12}}{{\bar{y}}_{\left(st\right)}\sqrt{\lambda (\frac{1}{2})}\lambda }]}>0$$
- By taking (9) and (24),
$${MSE({\hat{\bar{Y}}}_{ss(st)})}_{min}<MSE({\hat{\bar{Y}}}_{BT,P(st)})\mathrm{if}$$
$$MSE({\hat{\bar{Y}}}_{BT,P(st)})‐{MSE({\hat{\bar{Y}}}_{ss(st)})}_{min}>0$$
$$\frac{{\bar{Y}}^{2}[\frac{\left(-\frac{1}{4}v_{020}-v_{110}-v_{200})\lambda ({Ɲ}_{12}\right)}{\sqrt{\lambda (\frac{1}{2})}}+v_{200}\left[{Ɲ}_{13}\right]]}{\lambda [\frac{{Ɲ}_{12}}{\sqrt{\lambda (\frac{1}{2})}\lambda }]}>0$$
- By taking (11) and (24),
$${MSE({\hat{\bar{Y}}}_{ss(st)})}_{min}<{Var({\hat{\bar{Y}}}_{dif(st)})}_{min}\;if$$
$${Var({\hat{\bar{Y}}}_{dif(st)})}_{min}-{MSE({\hat{\bar{Y}}}_{ss(st)})}_{min}>0$$
$$\frac{{\bar{Y}}^{2}[\frac{\left(-v_{020}v_{110}+v_{110})\lambda ({Ɲ}_{12}\right)}{\sqrt{\lambda (\frac{1}{2})}}+v_{200}\left[{Ɲ}_{13}\right]]}{\left[\frac{{Ɲ}_{12}}{\sqrt{\lambda (\frac{1}{2})}}\right]}>0$$
- By taking (13) and (24),
$${MSE({\hat{\bar{Y}}}_{ss(st)})}_{min}<{MSE({\hat{\bar{Y}}}_{R,D(st)})}_{min}\;if$$
$${MSE({\hat{\bar{Y}}}_{R,D(st)})}_{min}-{MSE({\hat{\bar{Y}}}_{ss(st)})}_{min}>0$$
$$\frac{{\bar{X}}^{2}[\frac{\left(v_{020}v_{200}-{v_{110}}^{2})\lambda ({Ɲ}_{12}\right)}{\sqrt{\lambda (\frac{1}{2})}}-(v_{200}+1)v_{020}-{v_{110}}^{2}\left[{Ɲ}_{13}\right]]}{\left((v_{200}+1)v_{020}-{v_{110}}^{2})[\frac{{Ɲ}_{12}}{\sqrt{\lambda (\frac{1}{2})}}\right]}>0$$
- By taking (15) and (24),
$${MSE({\hat{\bar{Y}}}_{ss(st)})}_{min}<{\hat{\bar{Y}}}_{Singh(st)}\;\mathrm{if}$$
$${\hat{\bar{Y}}}_{Singh(st)}‐{MSE({\hat{\bar{Y}}}_{ss(st)})}_{min}>0.$$
$$\frac{{\bar{Y}}^{2}[\frac{\left(-\frac{1}{4}v_{020}+v_{110}-v_{200})\lambda ({Ɲ}_{12}\right)}{\sqrt{\lambda (\frac{1}{2})}\lambda }+v_{200}\left[{Ɲ}_{13}\right]]}{\lambda [\frac{{Ɲ}_{12}}{\sqrt{\lambda (\frac{1}{2})}\lambda }]}>0$$
- By taking (18) and (24),
$${MSE({\hat{\bar{Y}}}_{ss(st)})}_{min}<{{\hat{\bar{Y}}}_{GK(st)}}_{min}\;\mathrm{if}$$
$${{\hat{\bar{Y}}}_{GK(st)}}_{min}‐{MSE({\hat{\bar{Y}}}_{ss(st)})}_{min}>0$$
$$\frac{\frac{1}{64}[\frac{\left({-X}^{2}(16v_{020}-64)(-{v_{110}}^{2}+v_{200}+1)\lambda ({Ɲ}_{12}\right)}{\sqrt{\lambda (\frac{1}{2})}}-v_{200}\left[{Ɲ}_{13}\right]]}{\left[v_{020}(-{v_{110}}^{2}+v_{200}+1)]\lambda [\frac{{Ɲ}_{12}}{\sqrt{\lambda (\frac{1}{2})}}\right]}>0$$
- By taking (21) and (24),
$${MSE({\hat{\bar{Y}}}_{ss(st)})}_{min}<{MSE({\hat{\tilde{Y}}}_{Pr_{st}})}_{min}\;\mathrm{if}$$
$${MSE({\hat{\tilde{Y}}}_{Pr_{st}})}_{min}‐{MSE({\hat{\bar{Y}}}_{ss(st)})}_{min}>0$$
$$\frac{{\bar{Y}}^{2}\{64v_{200}(1-R_{y.xrx}^{2})-{v_{020}}^{2}-16v_{020}v_{200}(1-R_{y.xrx}^{2})\}}{64\{1+v_{200}(1-R_{y.xrx}^{2})\}}$$
$$‐\frac{{\bar{Y}}^{2}v_{200}\{T(\lambda )-B_{11}(\lambda )+v_{020}+4(R_{yx}^{2}-1)\}}{\left\{\lambda (4\frac{v_{200}}{\lambda }(R_{yx}^{2}-1)+4\frac{v_{020}^{1/2}}{\lambda^{1/2}}(S_{11}+B_{11})+T+\frac{v_{020}}{\lambda }-4)\right\}}>0.$$

## 5. Numerical investigation

In this portion, we run a numerical test to see how well the existing and suggested estimator performed; two populations are considered for this purpose. To get the PREs, we utilize the below expression:

$$PRE(.)=\frac{Var({\hat{\bar{Y}}}_{\left(st\right)})}{MSE({\hat{\bar{Y}}}_{i(st)})}*100,$$

where i = (${\hat{\bar{Y}}}_{R},{\hat{\bar{Y}}}_{P},{\hat{\bar{Y}}}_{BT,R},{\hat{\bar{Y}}}_{BT,P},{\hat{\bar{Y}}}_{dif},{\hat{\bar{Y}}}_{R,D},{\hat{\bar{Y}}}_{Singh},{\hat{\bar{Y}}}_{Gk},{\hat{\tilde{Y}}}_{Pr_{st}},{\hat{\bar{Y}}}_{ss}$).

## 6. Data discription

To show the efficiancy of proposed estimator over the existing estimators, we conduct a numerical study to investigate the performances of the propose and existing estimator. For this purpose, we used two real data sets of Kadilar and Cingi (2003), summary statistics given in Tables 1 and 2 for the population-I while MSE and PRE are presented in Table 3, similarly summary statistics for population-II given in Tables 4 and 5 while MSE and PRE are presented in Table 6. For population-I study variable is apple production in 1999, and the auxiliary variable is the number of apple trees in 1999. Similarly for population-II the study variable is apple production in 1999, and the auxiliary variable is the number of apple timber in 1998. (Source: Institute of Statistics, Republic of Turkey). We have stratified the data by regions of Turkey such as (1: Marmara, 2: Agean, 3: Miditerranean, 4: Central Anatolia, 5: Black sea, 6: East and Southeast Anatolia) and from each stratum, we have randomly selected the samples whose sizes are computed by using the Neyman allocation method.

**Table 1:** Summary statistics of Population-I.

H	Ŋ_h_	*n*_*h*_	ɷ_*h*_	*λ*_*h*_	${\bar{Y}}_{h}$	$S_{yh}^{2}$	$C_{yh}^{2}$	*β*_*yh*_
C 1	106	9	0.1241	0.1017	1536.774	41281746	17.479	79.340
2	106	17	0.1241	0.0494	2212.594	133437791	27.256	96.075
3	94	38	0.1100	0.0157	9384.309	894457433	10.156	25.809
4	171	67	0.2002	0.0090	5588.012	820445636	26.274	101.979
5	204	7	0.2389	0.1379	966.955	5710999	6.107	55.051
6	173	2	0.2026	0.4942	404.398	894440.3	5.469	30.119
${\bar{X}}_{h}$	$S_{xh}^{2}$	$C_{xh}^{2}$	*β*_*xh*_	*S*_*xh*_	${\bar{R}}_{xh}$	$S_{rxh}^{2}$	$C_{rxh}^{2}$	*β*_*rxh*_
24711.81	2414224935	3.95	27.46	49134.7	53.5	945.15	0.33	27.46
26840.04	2913701588	4.04	31.98	53978.7	53.5	945.04	0.33	31.98
72723.76	25956279019	4.90	27.40	161109.5	47.5	744.13	0.32	27.40
73191.2	68903936687	12.86	86.30	262495.6	86	2450.96	0.33	86.30
26833.75	2040714047	2.83	29.72	45174.2	102.5	3484.90	0.33	29.72
9903.30	360137210	3.67	31.87	18977.2	87	2508.36	0.33	31.87

**Table 2:** Correlation and covariance’s using Population I.

$\rho_{y_{h}x_{h}}$	$S_{y_{h}x_{h}}$	$\rho_{y_{h}{rx}_{h}}$	$S_{y_{h}{rx}_{h}}$	$\rho_{x_{h}{rx}_{h}}$	$S_{x_{h}{rx}_{h}}$
0.815688	257508714	0.3346206	66097.3	0.5956644	899792.9
0.835927	521230984	0.2814818	99957.52	0.6246736	1036577
0.897193	4323018145	0.4626493	377448.3	0.5893881	2590289
0.981495	7379640297	0.2979733	422543.1	0.3885082	5048833
0.710732	76727855	0.4541102	64063.84	0.6317101	1684630
0.869703	15609193	0.5366774	25420.49	0.6283556	597220.2

**Table 3:** MSE and PRE using Population I.

Estimators	MSE	PRE
${\hat{\bar{Y}}}_{\left(st\right)}$	697789.6	100
${\hat{\bar{Y}}}_{R(st)}$	243316.9	286.78
${\hat{\bar{Y}}}_{P(st)}$	1878241	37.15
${\hat{\bar{Y}}}_{BT,R(st)}$	379805.9	183.72
${\hat{\bar{Y}}}_{BT,P(st)}$	1197268	58.28
${\hat{\bar{Y}}}_{dif(st)}$	237552.8	293.74
${\hat{\bar{Y}}}_{R,D(st)}$	231157	301.87
${\hat{\bar{Y}}}_{Singh(st)}$	379805.9	183.72
${\hat{\bar{Y}}}_{GK(st)}$	228480.4	305.4
${\hat{\tilde{Y}}}_{Pr_{st}}$	207876.3	335.68
${\hat{\bar{Y}}}_{ss(st)}$	194803.2	358.2

**Table 4:** Summary statistics of Population II.

H	Ŋ_*h*_	*n*_*h*_	ɷ_*h*_	*λ*_*h*_	${\bar{Y}}_{h}$	$S_{yh}^{2}$	$C_{yh}^{2}$	$\beta_{yh}$
1	106	9	0.1241	0.1017	1536.774	41281746	17.479	79.340
2	106	17	0.1241	0.0494	2212.594	133437791	27.256	96.075
3	94	38	0.1100	0.0157	9384.309	894457433	10.156	25.809
4	171	67	0.2002	0.0090	5588.012	820445636	26.274	101.979
5	204	7	0.2389	0.1379	966.955	5710999	6.107	55.051
6	173	2	0.2026	0.4942	404.398	894440.3	5.469	30.119
${\bar{X}}_{h}$	$S_{xh}^{2}$	$C_{xh}^{2}$	*β*_*xh*_	*S*_*xh*_	${\bar{R}}_{xh}$	$S_{rxh}^{2}$	$C_{rxh}^{2}$	$\beta_{rxh}$
24375.59	2419565835	4.0	27.45	49189.0	53.5	945.142	0.3302	27.45607
27421.70	3301722268	4.3	35.90	57460.6	53.5	945.057	0.3301	35.90683
72409.95	25842911825	4.9	27.70	160757.3	47.5	744.139	0.3298	27.7032
74364.68	81569146488	14.7	97.73	285603.1	86	2450.935	0.3313	97.7351
26441.72	2061412416	2.9	29.77	45402.7	102.5	3484.892	0.3316	29.77505
9843.82	353212773	3.6	30.26	18793.9	87	2508.387	0.3314	30.26428

**Table 5:** Correlation and covariance’s using Population II.

$\rho_{y_{h}x_{h}}$	$S_{y_{h}x_{h}}$	$\rho_{y_{h}{rx}_{h}}$	$S_{y_{h}{rx}_{h}}$	$\rho_{x_{h}{rx}_{h}}$	$S_{x_{h}{rx}_{h}}$
0.8156414	257778692	0.3349042	66152.81	0.593033	896801.5
0.8559991	568176176	0.281644	100015.9	0.6031022	1065345
0.9011201	4332446622	0.4637681	378362.4	0.5873879	2575864
0.9858761	8065108356	0.2981778	422830.3	0.3654157	5166734
0.7130988	77372777	0.4547748	64157.44	0.6206547	1663517
0.8935989	15883145	0.5435647	25746.85	0.6262896	589509.3

**Table 6:** MSE and PRE using Population II.

Estimators	MSE	PRE
${\hat{\bar{Y}}}_{\left(st\right)}$	697789.6	100
${\hat{\bar{Y}}}_{R(st)}$	196709.7	354.73
${\hat{\bar{Y}}}_{P(st)}$	1924848	36.25
${\hat{\bar{Y}}}_{BT,R(st)}$	356502.2	195.73
${\hat{\bar{Y}}}_{BT,P(st)}$	1220572	57.17
${\hat{\bar{Y}}}_{dif(st)}$	183576.4	380.11
${\hat{\bar{Y}}}_{R,D(st)}$	179733.4	388.24
${\hat{\bar{Y}}}_{Singh(st)}$	356502.2	195.73
${\hat{\bar{Y}}}_{GK(st)}$	177598.9	392.9
${\hat{\tilde{Y}}}_{Pr_{st}}$	152565.6	457.37
${\hat{\bar{Y}}}_{ss(st)}$	140498.5	496.65

**Population I:** (Source: Kadilar and Cingi [18])

Y is the crop of apples in 1999, and X is the number of apples timber in 1999.

**Population 2:** (Source: Kadilar and Cingi [18])

Y is the crop of apples in 1999, and X is the crop of apples trees in 1998.

### 7. Simulation study

The efficiency of proposed estimators over competing estimators was demonstrated clearly in the preceding section. A Monte Carlo simulation analysis with R software is also used to assess the efficiency of the proposed estimator using dual ancillary variable under stratified random sampling. The assessment of proposed estimator with existing estimators is illustrated using the percentage relative efficiency (PRE) formula. Yet again, the real population of Kadilar and Cingi [18] is used. The following steps are used in R-Language software to conduct the simulation study.

1. We considered different sample sizes (64, 80, 96, 128, 144, 160, 176, 192, 208, and 240) through the proportional allocation method.
2. With stratified sampling, the technique is repeated 100,000 times and the population is divided into six strata to calculate the numerous values of proposed and existing estimators.
3. The 100,000 values of existing estimators and proposed estimator, as well as their corresponding variances, are computed using the samples obtained.
4. The values of percentage relative efficiency (PRE) are derived using the values of variances of all existing and proposed estimator and provided in Table 7.

**Table 7:** Simulation results for the PRE of recommended estimator w.r.t the existing estimators by different sample sizes.

Estimator	Sample Size									
	64	80	96	128	144	160	176	192	208	240
${\hat{\bar{Y}}}_{\left(st\right)}$	100	100	100	100	100	100	100	100	100	100
${\hat{\bar{Y}}}_{R(st)}$	375.87	380.11	387.46	389.17	385.078	380.028	390.00	410.00	425.00	542.001
${\hat{\bar{Y}}}_{P(st)}$	45.87	48.50	49.73	51.43	59.56	69.164	60.850	66.24	77.53	88.34
${\hat{\bar{Y}}}_{BT,R(st)}$	199.87	210.98	215.02	216.15	223.026	225.65	226.25	228.11	230.024	234.371
${\hat{\bar{Y}}}_{BT,P(st)}$	60.87	62.15	63.44	66.74	67.026	68.65	69.25	69.11	72.024	75.371
${\hat{\bar{Y}}}_{dif(st)}$	382.87	398.11	399.12	400.46	410.028	416.00	419.00	437.001	467.00	489.00
${\hat{\bar{Y}}}_{R,D(st)}$	390.87	395.50	399.73	415.850	416.24	419.53	426.34	428.15,	460.29	499.43
${\hat{\bar{Y}}}_{Singh(st)}$	199.87	210.98	215.02	216.15	223.026	225.65	226.25	228.11	230.024	234.371
${\hat{\bar{Y}}}_{GK(st)}$	399.49	411.76	423.76	445.56	456.78	459.12	467.23	489.78	490.34	499.89
${\hat{\tilde{Y}}}_{Pr_{st}}$	470.87	479.11	,489.12	498.46	499.17	494.078	512.028	517.00	519.00	520.001
${\hat{\bar{Y}}}_{ss(st)}$	518.87	520.50	523.73	565.43	586.56	596.16	598.850	599.24	605.53	647.34

The consequence of the above results, the performance of the proposed estimator is the best among all the existing estimators under consideration.

### Advantages of Monte Carlo simulation

The basis of a Monte Carlo simulation is that the probability of varying outcomes cannot be determined because of random variable interference. Therefore, a Monte Carlo simulation focuses on constantly repeating random samples to achieve certain results.

A Monte Carlo simulation takes variable that has uncertainty and assigns it a random value. The model is then run, and a result is provided. The process is repeated again and again while assigning the variable is question with many different values. Once the simulation is complete, the results are averaged together to provide an estimate.

## 8. Discussion

To evaluate the advantage of our propose estimator under stratified random sampling, we use two real data sets for numerical comparision. On the basis of numerical results, which are presented in Tables 3 and 6, it is observed that the proposed ratio-in-regression type estimator are more efficient than the usual sample mean estimator, Cochran [24], Murthy [25], Bahl and Tuteja [26], difference estimator, Rao [27], Singh et al. [28], Grover and Kaur [29], Ahmad et al. [30].

Table 7 gives simulation results for the percentage relative efficiency of proposed estimator w.r.t the existing estimators by using different sample sizes i.e: 64, 80, 96, 128, 144, 160, 176, 192, 208, and 240. The value of percentage relative efficiency differs depending on the sample size. From the simulation results, it is also observed that the proposed estimator is more efficient than the existing counterparts, in terms of percentage relative efficiency. As we increase the sample size, the efficiency of our proposed estimator is also increased. Overall, the gain in efficiency of our proposed estimator is the best as compared to all existing counterparts.

**Fig 1:**
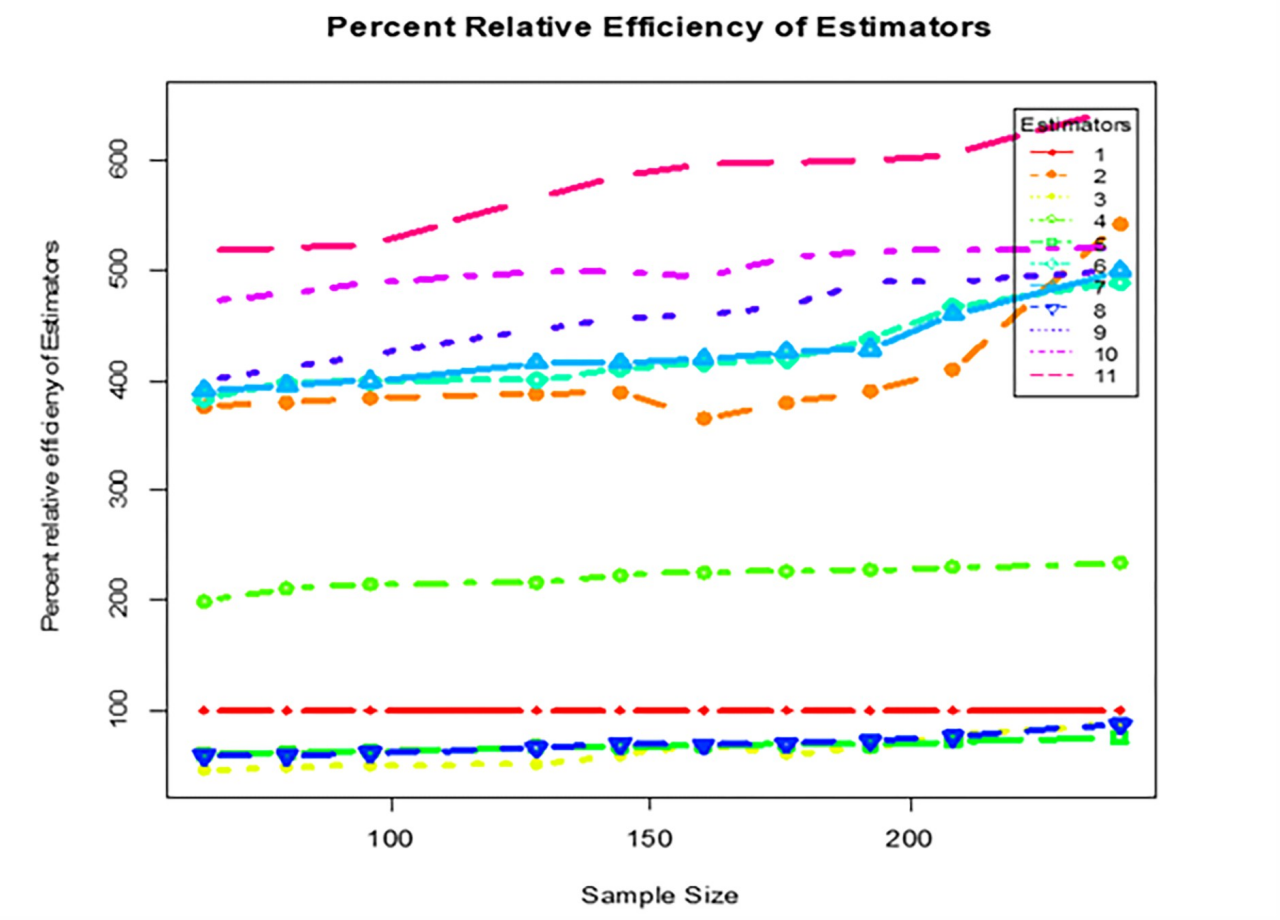
Percentage relative efficiency of different estimators.

For visualization, the comparison of proposed estimator with existing estimators in terms of percentage relative efficiency are presented in Fig 1. The length of a line graph is directly associated with the efficiency of an estimator. More specifically, the higher the length of a line graph, efficient the estimator. In general, we recommend using our proposed estimate for the new survey instead of the existing estimator examined in this paper for estimating the finite population mean under stratified random sampling.

## 9. Conclusion

In this article, we propose ratio-in-regression type exponential estimator for the finite population mean under stratified random sampling, which required an ancillary variable on the sample mean and rank of the ancillary varaible. Expressions for mean square error of the proposed estimator are derived up to first order of approximation and comparison is made with the estimators mentioned herein. According to results of real data sets, it is perceived that the proposed estimator performs well as compared to its existing counterpart. A simulation analysis is also carried out to assess the robustness and generalizability of the propose estimator. The simulation study’s findings also confirm the utility of the proposed estimator. A numerical study is carried out to support the theoretical results. Therefore we recommend the use of proposed estimators for efficiently estimating the finite population mean under stratified random sampling. The current work can be extended to develop an improved class of estimators under two-phase, non-response, two-stage, and cumulative distribution function sampling scheme using information on ancillary variable for estimating the population mean under simple and stratified random sampling.
